# A Novel Poly(vinyl alcohol)–tetraethylorthosilicate Hybrid Gel Electrolyte for Lead Storage Battery

**DOI:** 10.3390/gels8120791

**Published:** 2022-12-02

**Authors:** Bipin S. Chikkatti, Ashok M. Sajjan, Prakash B. Kalahal, Nagaraj R. Banapurmath, T. M. Yunus Khan, Shaik Dawood Abdul Khadar, Shaik Mohamed Shamsudeen, A. B. Raju

**Affiliations:** 1 Department of Chemistry, KLE Technological University, Hubballi 580031, India; 2 Centre of Excellence in Material Science, KLE Technological University, Hubballi 580031, India; 3 Department of Mechanical Engineering, College of Engineering, King Khalid University, Abha 61421, Saudi Arabia; 4 Industrial Engineering Department, College of Engineering, King Khalid University, Abha 61421, Saudi Arabia; 5 Department of Diagnostic Dental Science and Oral Biology, College of Dentistry, King Khalid University, Abha 61421, Saudi Arabia; 6 Department of Electrical and Electronics Engineering, KLE Technological University, Hubballi 580031, India

**Keywords:** polymer gel electrolyte, VRLA battery, cyclic voltammetry, electrochemical impedance spectroscopy, galvanostatic charge–discharge

## Abstract

The gel electrolyte significantly influences gel valve-regulated lead acid battery performance. To address this, the paper describes the preparation of novel polymer gel electrolytes using poly (vinyl alcohol) (PVA) and tetraethylorthosilicate (TEOS) for valve-regulated lead–acid batteries. FTIR technique is used to confirm the chemical reaction between PVA and TEOS. Electrochemical analyses such as cyclic voltammetry and electrochemical impedance spectroscopy were applied to optimize the concentration of PVA-TEOS polymer gel electrolyte. The optimum concentration of polymer gel electrolyte was determined as 20 wt% of TEOS in PVA (PE-1) with higher anodic peak and lower Rs and Rct values. The Galvanostatic charge–discharge tests were performed on the optimized gel system prototype battery. The highest capacity of 6.86 × 10^−5^ Ah at a current density of 0.2 mA cm^−2^ was achieved with an excellent capacity retention ratio of 85.7% over 500 cycles. The exceptional cycle performance and high capacity make PVA-TEOS gel electrolyte a promising candidate for practical battery application.

## 1. Introduction

The need for energy in today’s knowledge-based societies is growing, and energy storage technologies are becoming increasingly important [1]. Although traditional energy sources such as coal and petroleum generate the majority of the needed energy, their negative impacts on the environment and ecosystem have become a significant issue for the planet [2]. This issue can be resolved by electrochemical energy storage systems by effectively storing the produced energy in the chemical form [3]. Currently, batteries are being improved further to power a growing number of uses, including portable devices, electric cars, and smart grids [4]. The essential energy storage technologies today are valve-regulated lead–acid (VRLA) batteries, which were initially introduced in the early 1970s. Due to their benefits, including great energy efficiency, low cost, and extended cyclic life, VRLA batteries have a wide range of industrial applications, including the automobile industry, portable energy systems, and others [5]. Although interest in VRLA batteries has grown over the past two decades, their performance still has to be significantly improved [6,7]. Common lead–acid battery types include the following: batteries with excess or flooded electrolyte, low maintenance lead–acid batteries with a significant amount of excess electrolyte, and absorptive glass–microfibre (AGM) valve-regulated lead–acid (VRLA) batteries with immobilized electrolyte [8,9]. Electrodes, membranes, and electrolytes are the three primary components of VRLA battery systems. Each component has a big impact on the system’s capability and cyclical life. In terms of the electrolyte component, researchers have created two basic electrolyte technologies, including gel electrolyte and absorbed glass mat (AGM) systems. In an AGM electrolyte system, sulfuric acid is adsorbed onto a certain type of glass mat, and this system is known as an AGM VRLA battery. The gelled electrolyte system, known as the GEL–VRLA battery, is created by combining the gelling agent with the proper concentration of sulfuric acid. Particularly when used at low and high temperatures, the gel electrolyte system performs better than the AGM electrolyte system. AGM and flooded-type lead acid batteries are more impacted by operating temperature than gel type lead–acid batteries [10].

In general, gels are described as polymers and their swollen materials with three-dimensional network structures that are insoluble in any solvent and exist under peculiar conditions not found in solids, liquids, and gases. Polymer gels are comprised of a polymer network and solvents; the polymer network encloses the liquid and prevents it from escaping, or, in other words, acts as a container to hold a lot of solvents, giving it properties of both liquids and solids [11]. Gels often have high mobility because the polymer networks are solvated by a significant portion of the trapped solvent. The replacement of the solvent by liquid electrolyte having high value of conductivity results in polymer gel electrolytes [12]. The gelled electrolyte is one of the main elements influencing the quality of function of gel–VRLA batteries. During the creation of the gel, several variables, including the concentration of the sulphuric acid solution and the kind and concentration of gelling agents, can affect the properties of the gelled electrolyte such as the gel strength and rheology. In turn, the gelled electrolyte characteristics impact the electrolyte filling procedure, which in turn impacts the performance of the gel–VRLA batteries [13].

Admirable mechanical characteristics and high ionic conductivity are the requirements that gel electrolytes must generally meet, and these requirements call for proper tuning of the gel electrolyte components. A lot of work has been put into the electrochemical production of polymer gel electrolytes during the past few decades. Due to their distinct qualities such as facile moulding, good electrode–electrolyte interaction, and light weight, polymer electrolytes have emerged as a material of major relevance for many electrochemical devices [14]. In recent years, a number of polymer matrix materials, including poly (vinyl alcohol) (PVA) [15], poly (ethylene oxide) (PEO) [16], poly (methyl methacrylate) (PMMA) [17], poly (vinylidene fluoride) (PVDF) [18], and poly (acrylonitrile) (PAN) [19] have been studied for the preparation of gel electrolytes. Awadhia et al. reported that poly(vinyl alcohol) (PVA) can be used as a gel electrolyte because of its outstanding ion transport capability, good mechanical property, excellent chemical stability, high water solubility, remarkable swelling ability, high moisture retention, non-toxicity, and good biocompatibility which makes it a great material for research [20,21].

Recent research has led to the development of polymer–silica hybrids with improved thermal and mechanical capabilities (due to the silica), higher flexibility (due to the presence of polymers), and numerous customized properties which have found use in a number of areas, including catalysis [22], adsorption [23], photonics [24], white light-emitting diodes [25], Quantum Dot Light Emitting Diodes [26] and pervaporation [27]. In reality, a silica oligomer is created at the very beginning, which later aggregates into a nano-SiO_2_ particle [28]. The sol–gel method may synthesize silica from a variety of precursors, but the most popular one is tetraethylorthosilicate (TEOS), which can be easily processed and possesses a relatively slow and controllable reaction rate [29]. The hydrolysis and condensation reactions of the tetraethylorthosilicate (TEOS) result in a silica network with siloxane linkages (Si-O-Si) in the bulk and silanol groups at the surface (Si–OH). The reactivity of silica is primarily caused by the latter [30]. In addition, SiO_2_ possesses a hydrophilic characteristic that allows it to absorb water more forcefully. The ions in the inorganic materials are firmly hydrogen bound to the water molecules [31]. Kim et al. reported that at high temperatures, composite material demonstrated more water uptake and improved cell efficiency [32]. Studies using TEOS have demonstrated that the addition of the TEOS additive causes the synthetic material water absorption capacity to rise even at high temperatures and low relative humidity [33].

In this work, an attempt is made to increase the performance of VRLA battery by developing a novel PVA-TEOS polymer gel electrolyte. The chemical reaction of the polymer gel electrolyte was studied by Fourier transform infrared spectroscopy (FTIR) and optimization ratios of the TEOS was studied by cyclic voltammetry (CV) and electrochemical impedance spectroscopic (EIS) methods for the first time. Galvanostatic charge–discharge (GCD) experiments were then utilized to analyze the charge–discharge behavior of optimized polymer gel electrolytes.

## 2. Results and Discussion

### 2.1. Physico-Chemical Characterization of Developed Polymer Gel Electrolytes

#### Fourier Transform Infrared Spectroscopy (FTIR)

**Figure 1:**
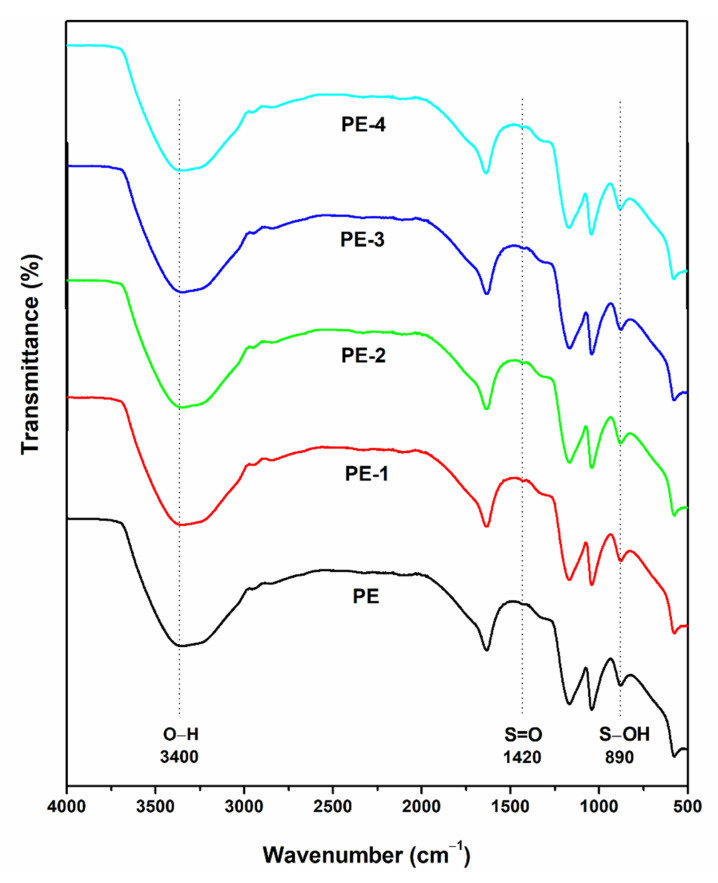
FTIR spectra of plane PVA (PE), PVA-20 wt% TEOS (PE-1), PVA-40 wt% TEOS (PE-2), PVA-60 wt% TEOS (PE-3) and PVA-80 wt% TEOS (PE-4) polymer gel electrolytes.

The incorporation of TEOS into PVA matrix was confirmed by FTIR studies. Figure 1 displays the FTIR spectra of plane PVA and those of various TEOS loadings. A characteristic strong and broad band appeared at 3400 cm^−1^ in plane PVA spectra (PE) corresponding to –OH stretching vibrations of the hydroxyl groups [34]. With increasing TEOS content, the intensity of this broadband gradually dropped from PE-1 to PE-4, indicating that some of the –OH groups of PVA were involved in a condensation reaction with the silanol groups of TEOS, resulting in the formation of covalently bound crosslinks between polymer segments.

Further multiple bands that appeared in the spectra (PE) at around 1000 and 1200 cm^−1^ were assigned to C-O stretching vibrations. The intensity of these bands increased marginally from PE-1 to PE-4 due to an increase in Si–O groups in the gels with increasing TEOS content, since the Si–O stretching band appears almost close to the frequency of C–O stretching that suggests the formation of Si-O-C bonds between the PVA and TEOS [35,36]. However, peaks at 1420 cm^−1^ and 890 cm^−1^ corresponds to S=O and S-OH stretching vibrations of H_2_SO_4_ in polymer gel electrolyte [37].

### 2.2. Electrochemical Performance of Developed Polymer Gel Electrolytes

#### 2.2.1. Cyclic Voltammetry (CV) Analysis

**Figure 2:**
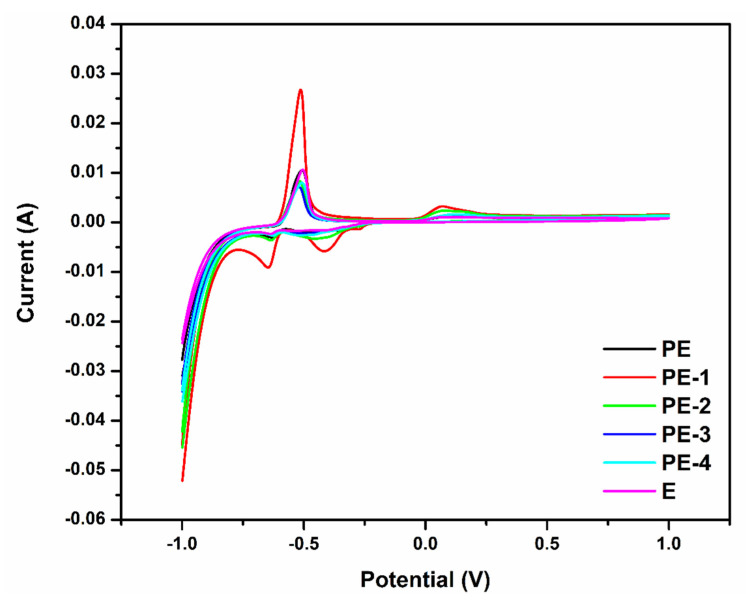
Cyclic voltammetry curves of plane PVA (PE), PVA-20 wt% TEOS (PE-1), PVA-40 wt% TEOS (PE-2), PVA-60 wt% TEOS (PE-3), PVA-80 wt% TEOS (PE-4) and 36 wt% H_2_SO_4_ (E) at a scan rate of 50 mV s^−1^.

Using lead as the working electrode and a scan rate of 50 mV s^−1^, the cyclic voltammetric behavior of produced polymer electrolytes was examined. The potential was scanned from −1 V to +1 V. Figure 2 represents cyclic voltammograms of PE, PE-1, PE-2, PE-3, PE-4, and E. The production of PbSO_4_ from a Pb electrode accounts for the oxidation peak at around −0.5 V. Peak potential for the reverse reduction process is at −0.4 V. In lead–acid batteries, the oxidation reaction represents the conversion of Pb to PbSO_4_ (discharge reaction) and reduction reaction represents the conversion of PbSO_4_ to Pb (charge reaction) (Equations (1) and (2)). For these reasons, CV analysis can be used to examine the fundamental characteristics of a lead–acid battery [38].

Pb + HSO_4_^−^ ↔ Pb^2+^ + SO_4_^2−^ + H^+^ + 2e^−^, (1)

Pb^2+^ + SO_4_^2−^ ↔ PbSO_4_. (2)

**Figure 3:**
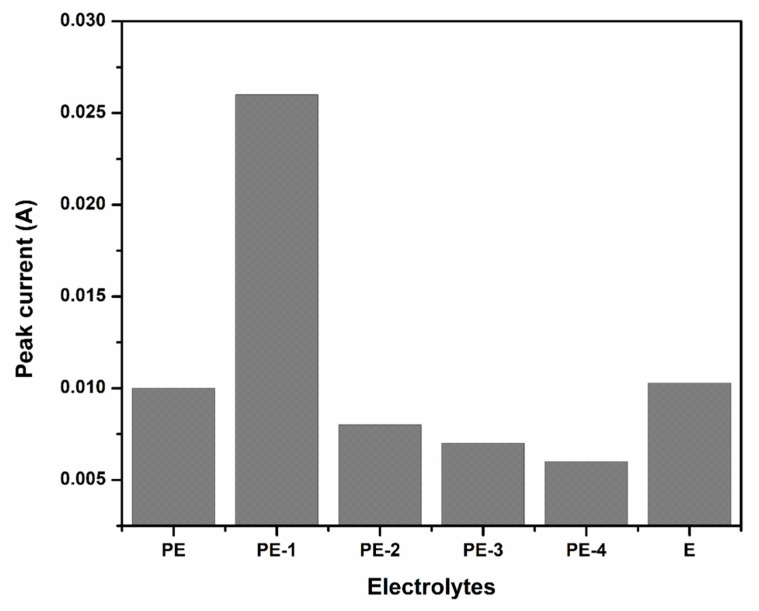
Anodic peak current values of plane PVA (PE), PVA-20 wt% TEOS (PE-1), PVA-40 wt% TEOS (PE-2), PVA-60 wt% TEOS (PE-3), PVA-80 wt% TEOS (PE-4) and 36 wt% H_2_SO_4_ (E) at a scan rate of 50 mV s^−1^.

The largest anodic peak current was identified in a PVA system with 20 wt% added TEOS. Since the reaction between the electrode surface and electrolyte ions occurred easier in this concentration than in others. The interactions of electrodes and mobile ions of electrolyte had the highest level according to obtained anodic and cathodic peak values. The result showed that using TEOS can increase the capacity and performance of the battery [39]. Further, the value of the anodic peak current was decreased from 40 wt% to 80 wt% of TEOS-containing gel electrolyte. Because of the deformation of the gel structure, the interactions of electrodes and electrolyte decrease, which results in a decrease in the anodic peak current value of the polymer gel electrolyte. Additionally, Figure 3 shows the change of peak current with various electrolytes.

**Figure 4:**
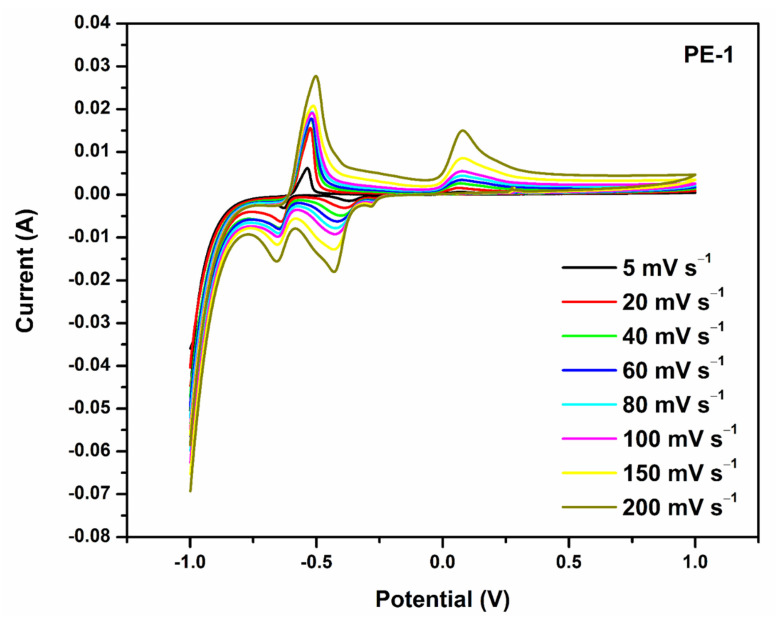
PVA-20 wt% TEOS (PE-1) cyclic voltammetric activity at numerous scan rates from 5 to 200 mV s^−1^.

**Figure 5:**
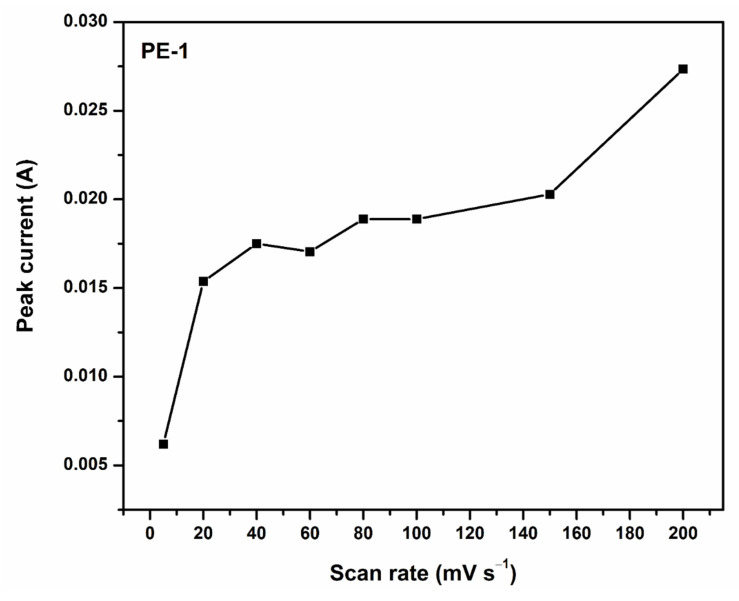
Anodic peak current values of PVA-20 wt% TEOS (PE-1) at various scan rates (5 to 200 mV s^−1^).

To determine the way in which the scan rate affected the values of the anodic peak current, the CV behavior of the optimized polymer gel electrolyte PVA-20 wt% TEOS (PE-1) was examined at numerous scan rates (5–200 mV s^−1^). From Figure 4 and Figure 5, it is observed that as the scan rate increased, the anodic peak current increased [40].

#### 2.2.2. Electrochemical Impedance Spectroscopy (EIS) Analysis

**Figure 6:**
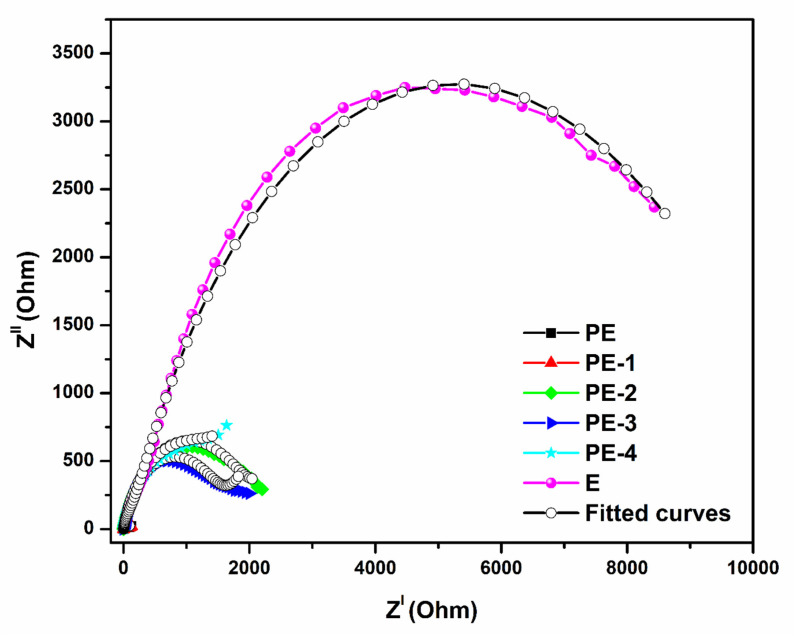
Electrochemical impedance spectra of plane PVA (PE), PVA-20 wt% TEOS (PE-1), PVA-40 wt% TEOS (PE-2), PVA-60 wt% TEOS (PE-3), PVA-80 wt% TEOS (PE-4) and 36 wt% H_2_SO_4_ (E).

Ion transport in the electrodes and the characteristics of the interface are both revealed by measurements from electrochemical impedance spectroscopy. The kinetic characteristics and the electrode reaction both affect the ability of an ion to move. The material morphology has a significant impact on these elements. Ion and electron transport processes are included in the order of transport, as well as the charge transfer process [41]. The Nyquist plot of the equivalent circuit fitted by the ZsimpWin software is shown in Figure 6. To simulate the impedance behavior of the polymer gel electrolyte and match the experimentally acquired impedance data, the equivalent circuit of model R(C(R(Q(RW)))) was utilized. The first one is the polymer and electrolyte bulk solution resistance (Rs), second one combines the double-layer capacitance (Cdl) with the electrolyte resistance (R_1_) in parallel. A series connection to electrolyte resistance (R_1_) is made up of using constant phase element (Q) in parallel with charge transfer resistance (Rct) and Warburg impedance (W) of the polymer gel electrolyte [42,43]. For 36% H_2_SO_4_ equivalent, circuit R(Q(R(QR)(Q(RW)))) was used.

**Figure 7:**
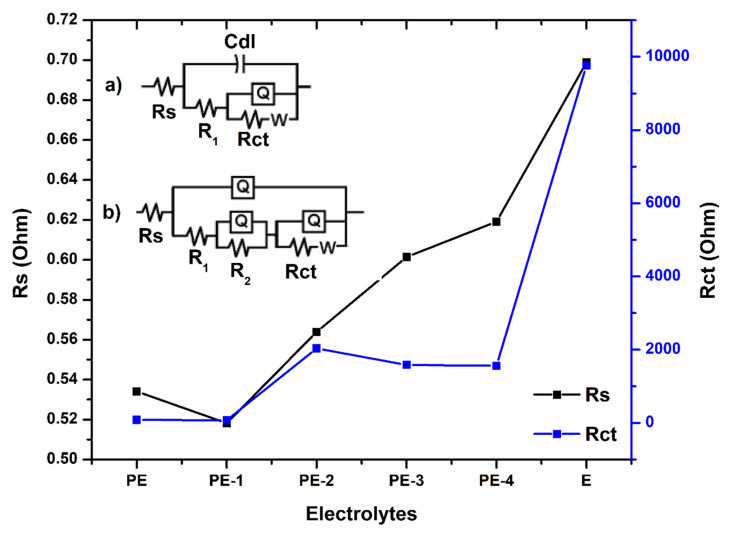
Equivalent circuits of (**a**) developed polymer gel electrolyte (**b**) 36 wt% H_2_SO_4_ and Solution resistance (Rs) and charge transfer resistance (Rct) of plane PVA (PE), PVA-20 wt% TEOS (PE-1), PVA-40 wt% TEOS (PE-2), PVA-60 wt% TEOS (PE-3), PVA-80 wt% TEOS (PE-4) and 36 wt% H_2_SO_4_ (E).

Rs and Rct parameters were studied in EIS spectra [10]. Figure 7 shows the Rs solution resistance and Rct charge transfer resistance values of electrolytes. The fitted impedance values of developed polymer gel electrolytes and 36 wt% H_2_SO_4_ are shown in Table 1 and Table 2.

Table 1 and Table 2 show that the Rs and Rct values are low for PVA-20 wt% TEOS (PE-1). Due to higher free ions in the polymer gel electrolyte, the mobility and conductivity of the polymer gel electrolyte were increased for PVA-20 wt% TEOS (PE-1), and electrode and electrolyte interaction must be there at the greatest level. Further, with the addition of TEOS beyond 20 wt%, higher Rs and Rct values were observed when compared to PVA-20 wt% TEOS (PE-1). This is due to the fact that restriction of the free ions leads to lower mobility of ions in the deformed three-dimensional structure of the developed polymer gel electrolyte [38].

#### 2.2.3. Galvanostatic Charge–Discharge (GCD) Analysis

**Figure 8:**
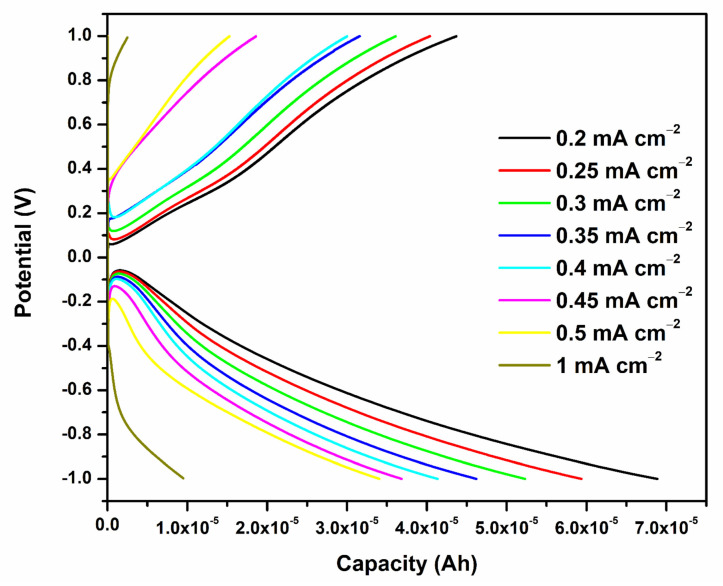
Charge–discharge curves of prototype battery with PVA-20 wt% TEOS (PE-1) at different current densities (0.2 to 1 mA cm^−2^).

Galvanostatic charge–discharge profiles of optimized PVA-20 wt% TEOS (PE-1) prototype battery test at variable current densities are shown in Figure 8, where the achieved capacity and the current densities are calculated.

Investigation of Figure 8 shows that the battery has a capacity of 6.86 × 10^−5^ Ah at 0.2 mA cm^−2^, with a quite low current density. When the charge–discharge current increases from 0.2, 0.25, 0.3, 0.35, 0.4, 0.45, 0.5, and 1 mA cm^−2^, the discharge capacity decreases from 6.86 × 10^−5^, 5.936 × 10^−5^, 5.229 × 10^−5^, 4.620 × 10^−5^, 4.135 × 10^−5^, 3.680 × 10^−5^, 3.405 × 10^−5^ and 9.5 × 10^−6^ Ah, respectively. The outcome shows that even at extremely high current densities, the PVA-20 wt% TEOS (PE-1) has great rate capability and structural integrity. This shows that a prototype battery with PVA-20 wt% TEOS (PE-1) can quickly charge and discharge and can adapt to the grid’s rapid changes in power supply and demand [4,44,45].

**Figure 9:**
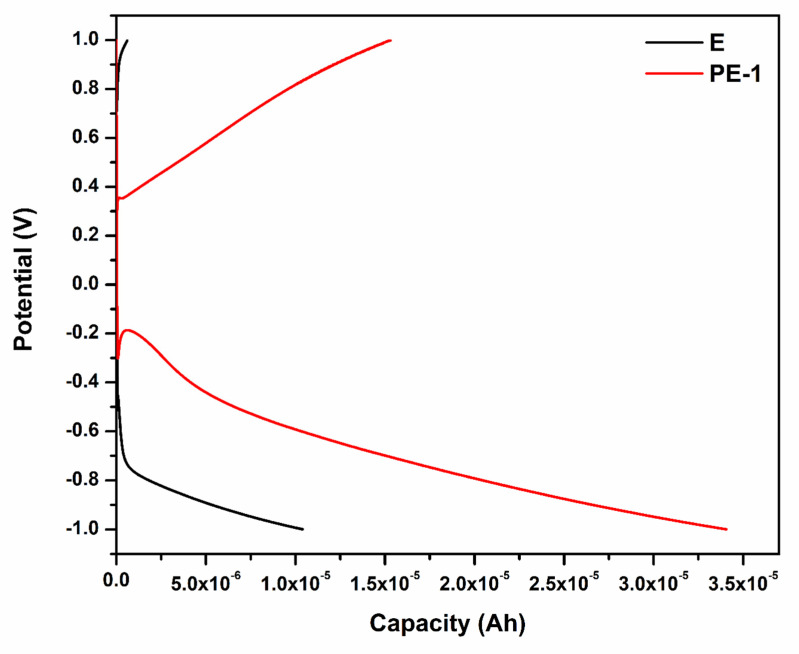
Charge–discharge curves of prototype battery with PVA-20 wt% TEOS (PE-1) and 36 wt% H_2_SO_4_ (E) at 0.5 mA cm^−2^ current density.

**Figure 10:**
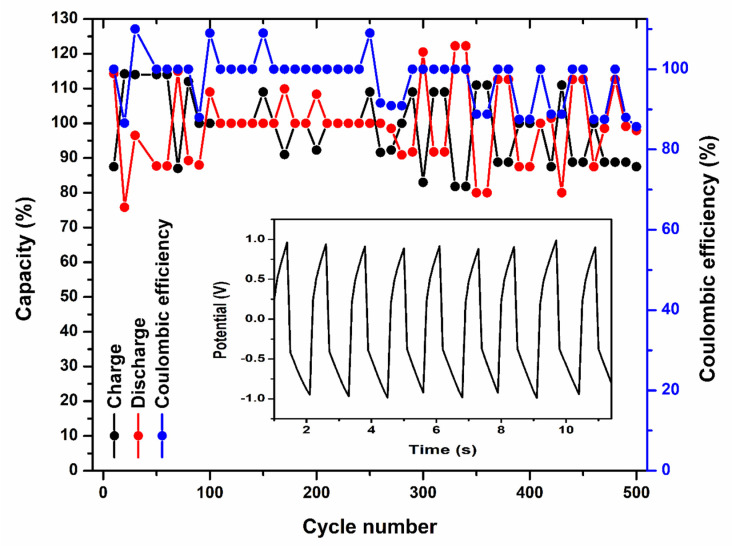
Prolonged cycle performance of prototype battery with PVA-20 wt% TEOS (PE-1) at a current density of 0.5 mA cm^−2^.

Figure 9 designates the charge and discharge curves of the prototype battery with PVA-20 wt% TEOS (PE-1) and 36 wt% H_2_SO_4_ (E) at 0.5 mA cm^−2^ current density. The prototype battery with PVA-20 wt% TEOS (PE-1) showed a capacity of 3.405 × 10^−5^ Ah, whereas 36 wt% H_2_SO_4_ (E) showed 1.036 × 10^−5^ Ah. The capacity of the battery with PVA-20 wt% TEOS (PE-1) is higher than that of 36 wt% H_2_SO_4_ (E). Figure 10 shows the cycle performance of the prototype battery with PVA-20 wt% TEOS (PE-1) at a current density of 0.5 mA cm^−2^. After 500 cycles, there was only a 14.3% retention of capacitance and coulombic efficiency of almost 100%, demonstrating remarkable cycle stability.

## 3. Conclusions

This study involved the preparation and characterization of a unique PVA-TEOS, a polymer gel electrolyte for gel–VRLA battery application. In FTIR, the increase in the intensity observed in the multiple peaks that appeared in the range between 1000 and 1100 cm^−1^ confirms the cross-linking reaction between PVA and TEOS. Further, the electrochemical performance of the polymer gel electrolyte was analyzed. Using the cyclic voltammetry technique, 20 wt% of TEOS in PVA (PE-1) is considered an optimized polymer gel electrolyte since it shows the highest anodic peak current at 50 mV s^−1^. From electrochemical impedance spectroscopy analysis, low Rs and Rct values were observed in 20 wt% of TEOS in PVA (PE-1). The battery performance of the optimized polymer gel electrolyte was analyzed using galvanostatic charge–discharge tests. In total, 20 wt% of TEOS in PVA (PE-1) exhibits exceptional cycle performance with a large capacity and better rate capability. The improved polymer gel electrolyte obtained a great capacity retention ratio of 85.7% over 500 cycles and delivered a capacity of 6.86 × 10^−5^ Ah at a current density of 0.2 mA cm^−2^. The prototype battery with PVA-20 wt% TEOS demonstrated a capacity of 3.405 × 10^−5^ Ah at 0.5 mA cm^−2^ current density compared to 36 wt% H_2_SO_4_ with 1.036 × 10^−5^ Ah. As a result, PVA-20 wt% TEOS produces superior battery performance compared to 36 wt% H_2_SO_4_. As a result, polymer gel electrolyte PVA-TEOS overcomes the ideal properties of electrolytes used in conventional lead acid batteries. Therefore, PVA-TEOS hybrid gel electrolyte can be used as an electrolyte in a lead storage battery for industrial applications.

## 4. Materials and Methods

### 4.1. Materials

Poly(vinyl alcohol) (Mw ~ 124,000) was procured from s.d. fine Chemicals Ltd., Mumbai, India. Tetraethylorthosilicate (Mw ~ 208.33) was purchased from Sigma-Aldrich Chemicals, Saint Louis, MO, USA. From Spectrum Reagent and Chemicals Pvt. Ltd. in Cochin, India, sulfuric acid was provided. Distilled water was used during the whole research work.

### 4.2. Development of Polymer Gel Electrolytes

**Figure 11:**
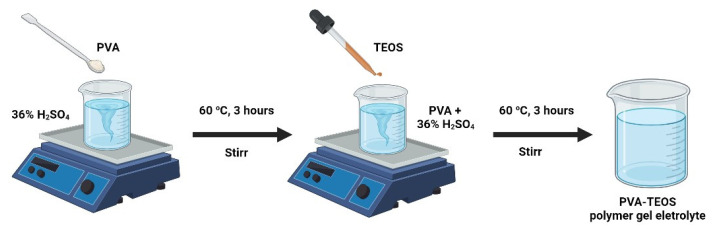
Diagram showing the process for making PVA-TEOS polymer gel electrolyte.

**Figure 12:**
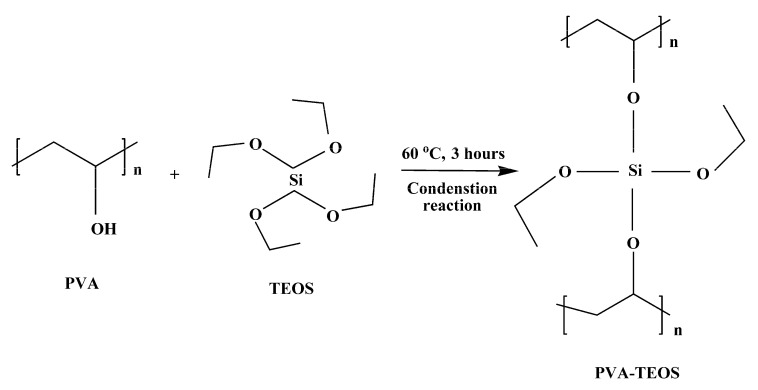
Possible scheme of interaction between PVA and TEOS.

First, 4 g of PVA and 100 mL of 36 wt% H_2_SO_4_ were combined, then stirred at 60 °C for 3 h. Filtered, the solution was given the PE designation. Then, to prepare a solution of PVA for the sol–gel reaction, the necessary amount of TEOS was added, and the outcome reaction solution was agitated for 3 h at 60 °C. Polymer gel electrolytes were formed by varying the amount of TEOS concerning PVA by 20, 40, 60, and 80 wt%. These electrolytes were labelled PE-1, PE-2, PE-3, and PE-4, respectively. For comparison, 36 wt% H_2_SO_4_ solution was prepared and represented as E. Figure 11 and Figure 12 show the PVA-TEOS polymer gel electrolyte preparation method and possible scheme of interaction between PVA and TEOS.

### 4.3. Physico-Chemical Characterization

The interactions between PVA and TEOS in the polymer gel electrolyte were investigated using Spectrum Two FTIR containing Diamond ATR (PerkinElmer Singapore Pvt. Ltd., 28, Ayer Rajah Crescent, no. 08-01, Singapore 139959). FTIR measurements were made between 500 and 4000 cm^−1^.

### 4.4. Electrochemical Performance

**Figure 13:**
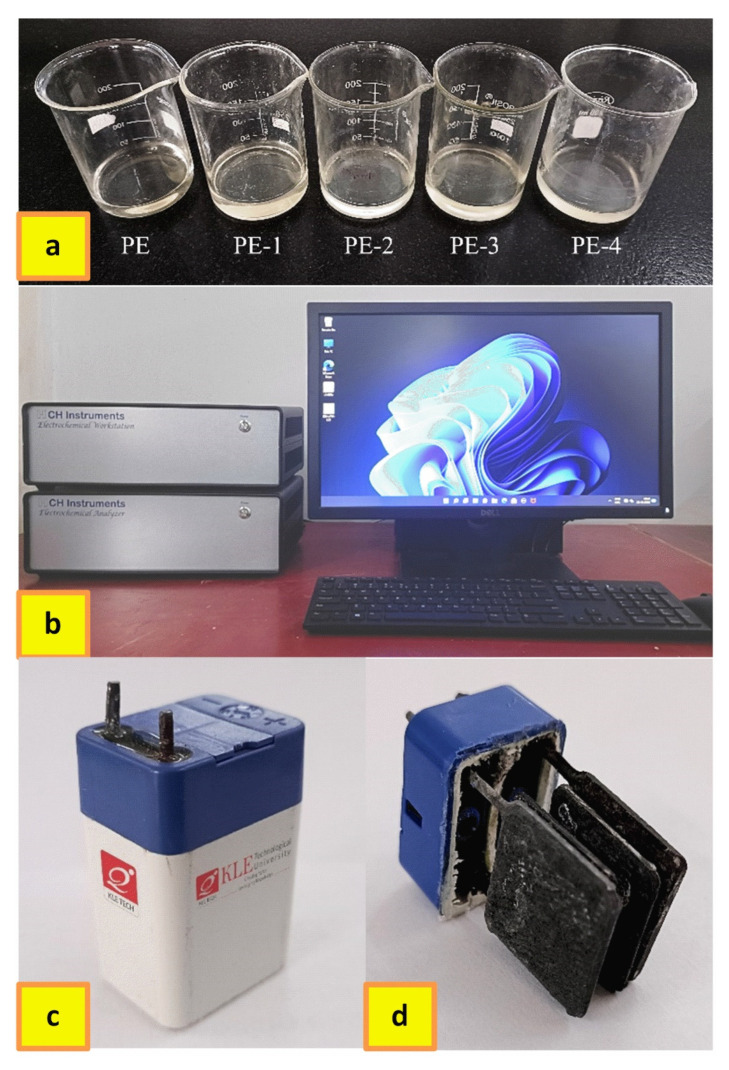
Photo image of (**a**) developed polymer gel electrolytes, (**b**) electrochemical workstation, (**c**) sealed prototype battery, (**d**) cross-section view of the battery showing electrodes.

Electrochemical characterization such as Cyclic voltammetry (CV), Electrochemical impedance spectroscopy (EIS), and Galvanostatic charge–discharge (GCD) of the developed polymer gel electrolytes was characterized using an electrochemical work station CHI660E, CH Instruments, Austin, TX, USA. During analysis, three-electrode systems were used for CV and EIS, with the lead being the working electrode (8 cm length, 6 mm diameter), Ag/AgCl, KCl (saturated) as a reference electrode, and platinum wire as the auxiliary electrode. Before every measurement, the working electrodes were polished. Cyclic voltammetry (CV) tests were conducted over the potential range from −1.0 to 1.0 V at a varying scan rate (5–200 mV s^−1^). The performance of samples was indicated by corresponding redox peak currents in the curves. Electrochemical impedance spectroscopy (EIS) experiments were carried out at a range of 100 kHz to 0.01 Hz frequency with 5 mV amplitude. The curves obtained enabled us to analyze the Solution resistance (Rs) and Charge transfer resistance (Rct). Galvanostatic charge–discharge (GCD) was performed using a two-electrode system in a cell with two negative electrodes, two positive electrodes, and 4.5 mL of synthesized gel electrolyte. The dimension of each electrode was 2 × 2 cm^2^. With various current densities, batteries were charged and discharged. All charge–discharge analyses were carried out between −1 and 1 V. All experiments were conducted at a temperature of 25 °C. Figure 13 shows photo images of developed polymer gel electrolytes, electrochemical workstation, sealed prototype battery, and cross-section view of the battery showing electrodes.

## Figures and Tables

**Table 1:** Fitted impedance values of plane PVA (PE), PVA-20 wt% TEOS (PE-1), PVA-40 wt% TEOS (PE-2), PVA-60 wt% TEOS (PE-3), PVA-80 wt% TEOS (PE-4).

**Polymer Gel Electrolytes**	**Rs (Ohm)**	**Cdl (F)**	**R_1_ (Ohm)**	**Q (S-sec^n)**	**n**	**Rct (Ohm)**	**W (S-sec^5)**
PE	0.5341	0.0002509	1.97	0.005817	0.5971	82.59	0.1447
PE-1	0.5182	0.0002258	1.859	0.007118	0.5288	72.33	0.22
PE-2	0.5639	0.00004742	8.848	0.000348	0.6661	2035	0.01576
PE-3	0.6014	0.00003007	13.27	0.000189	0.6616	1585	0.008965
PE-4	0.6191	0.0001082	1.831	0.001566	0.7558	1561	0.007856

**Table 2:** Fitted impedance values of 36 wt% H_2_SO_4_ (E).

**Rs (Ohm)**	**Q (S-sec^n)**	**n**	**R_1_**	**Q (S-sec^n)**	**n**	**R_2_**	**Q (S-sec^n)**	**n**	**Rct**	**W (S-sec^5)**
0.6988	0.00006129	0.8374	0.02246	2.502×10^−18^	0.7992	350.5	0.0001736	0.6981	9763	0.009552
